# Bilateral posterior fracture-dislocation of the shoulders: Two rare case reports and literature review

**DOI:** 10.1097/MD.0000000000022088

**Published:** 2020-09-04

**Authors:** Junwei Wu, Lianxin Li, Fengrui Wang, Shun Lu, Fanxiao Liu, Honglei Jia, Yongliang Yang, Fu Wang, Zhenhai Hao, Shihong Xu, Bomin Wang

**Affiliations:** Department of Orthopaedics, Shandong Provincial Hospital Affiliated to Shandong First Medical University, No. 324 JingWu Road, 250021, Jinan, Shandong, PR China.

**Keywords:** bilateral, open reduction, posterior fracture-dislocation, shoulder

## Abstract

**Rationale:**

Bilateral posterior fracture-dislocation of the shoulders occurs rarely and the diagnosis is often challenging. This injury is often missed or delayed on initial presentation, leading to continuous pain, disability, and rising medical costs. Timely diagnosis and proper treatment are very important to restore shoulder function.

**Patient concerns:**

Here we report 2 rare cases. Case 1 was a 53-year-old physical worker with severe pain and limited shoulder movement after an unexpected fall. Case 2 was a 55-year-old man with pain in upper limbs and shoulders after an electric shock.

**Diagnosis:**

Both of them were diagnosed as bilateral posterior fracture-dislocation of the shoulders by computed tomography (CT) scan.

**Intervention:**

After systematic preoperative evaluation, both of them were treated with open reduction and internal fixation.

**Outcomes:**

After 16 months follow-up, case 1 was pain-free in both shoulders. He had returned to full activity and was satisfied with his level of function. At 24 months follow-up, both shoulders of case 2 were painless and stable with acceptable range of motion and he was able to carry out daily activities.

**Lessons:**

Our case reports highlight that bilateral posterior fracture-dislocation of the shoulders is easy to be missed; one way to prevent missing diagnosis is to suspect cases with pain and limited external rotation, especially those with a history of seizures, electric shock, or severe trauma; appropriate history inquiry, physical examination, proper shoulder images are the key to correct diagnosis.

## 1 Introduction

The shoulder joint is the most commonly dislocated joint of the human body, with an incidence of 10 to 24 per 100,000 person years.^[1,2]^ However, most shoulder dislocations are anterior and posterior dislocation is a rare entity accounting for 3% to 5% of all shoulder dislocations,^[3,4]^ whereas fracture-dislocations are exceedingly rare.^[5,6]^ In the 1500 cases reported by Neer et al, only 0.9% was posterior fracture-dislocations.^[7,8]^

Diagnosis of this injury is often challenging, often mistaken for “frozen shoulder,” aortic dissection, or myocardial infarction, thus frequently missed or delayed in the emergency department (ED).^[5,6]^ The rate of misdiagnosis might exceed 50% at the first examination^[9–11]^ and Hawkins et al reported that 3 quarters of this injury was delayed in diagnosis, with an average interval of 1 year.^[12]^ Possible reasons include: insufficient cooperation of patients, lack of clear clinical symptoms, inappropriate imaging evaluation, insufficient familiarity of experts with the disease, etc.^[13]^ Long intervals between trauma and therapy may increase the risk of humeral head collapse, osteoarthritis, shoulder stiffness, joint contracture, and osteonecrosis. Therefore, timely and accurate recognition and appropriate treatment are essential for optimal shoulder function restoration.

In this study, we report 2 rare cases of bilateral posterior fracture-dislocation of the shoulders and discuss their pathogenesis, clinical manifestations, imaging methods, and treatment options in combination with relevant literature, so as to improve the clinicians’ understanding of the disease and ensure that it is not missed or delayed. This study was approved by the Institutional Review Board of Shandong Provincial Hospital Affiliated to Shandong First Medical University. Written informed consent for publication of case details and pictures was obtained from the 2 patients and their families prior to the study.

## 2 Case reports

### 2.1 Case 1

**Figure 1:**
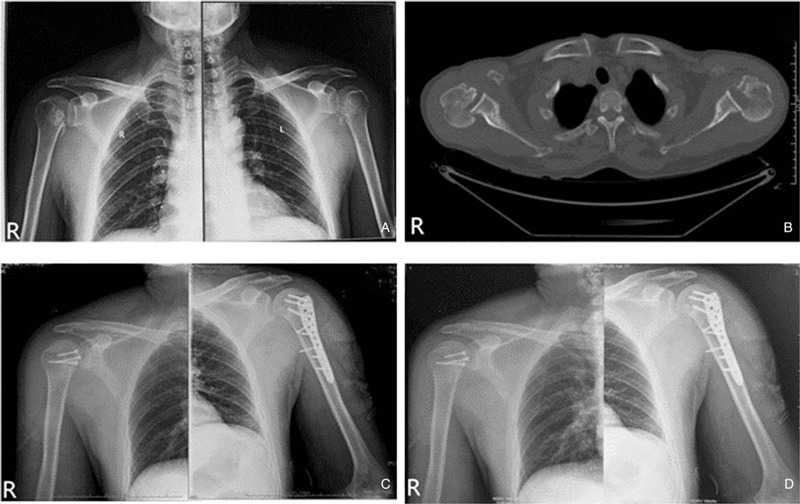
(a) Anteroposterior radiographs showing suspicious fractures of bilateral proximal humerus; (b) axial CT of both sides confirming the dislocation and showing a 3-part fracture on the left side and a 2-part fracture on the right side; (c) postoperative X-ray showing appropriate implants location; (d) X-ray findings after 16 months of follow-up showing good union and no avascular necrosis of the humeral head.

A 53-year-old physical worker presented with severe pain and restriction of movements involving both shoulders after an unexpected fall when he carried a large and heavy box through the uneven steps 15 days ago. He was conscious throughout the fall and had no history of epilepsy. Immediately after the injury, he tried to stand up and felt a sharp pain in his shoulders. Although he took analgesic, his pain continued unabated, and the movement of his shoulders was significantly limited. He turned to the local ED that afternoon. However, a routine anteroposterior radiograph of the shoulders was misinterpreted (Fig. 1a) and the dislocation was not diagnosed. Due to persistent pain and limited bilateral shoulder movements, he came to our ED on the next day and a traumatology assessment was conducted. Except for swelling and bruising of shoulder soft tissue, no obvious deformity was found in preliminary examination. The abnormality was that both arms of the patient were maintained in adduction and internal rotation and the shoulders cannot be rotated externally. Due to his muscularity, neither of his humeral heads could be palpable from his posterior shoulder. The detected neurovascular status of both upper limbs was intact. Bilateral posterior dislocation of shoulders was thus suspected. He was unable to cooperate with the axillary radiograph because of pain, so computed tomography (CT) scan was arranged, which showed a 3-part fracture on the left side and a 2-part fracture on the right side^[7]^ and he was diagnosed with bilateral posterior fracture-dislocation of the shoulder (Fig. 1b).

According to the AO fracture classification, the left side was 11-B3.3 fracture and the right side was 11-C1.3 fracture. He was quickly transferred to our orthopedics and traumatology for further treatment. After systematic preoperative evaluation, open reduction and internal fixation (ORIF) with a lock plate (Lateral Distal Humerus Plate II, Orthomed, China) was performed on the left side and ORIF with 2 cancellous bone screws (Cancellous Bone Screw, Orthomed, China) on the right side. Postoperative X-ray showed appropriate implants location (Fig. 1c). He was discharged 6 days postoperatively. Each shoulder was fixed in a sling for 3 weeks, followed by 3 weeks of passive motion. Active-assisted and pendulum exercises were started from the sixth week and formal strengthening was started from the twelfth week. After 16 months follow-up, he was pain-free in both shoulders. X-ray showed good union and there was no sign of avascular necrosis of the humeral head (Fig. 1d). The final Constant score^[14]^ was 90 for the right side and 88 for the left side. He had returned to full activity and was satisfied with his level of function.

### 2.2 Case 2

**Figure 2:**
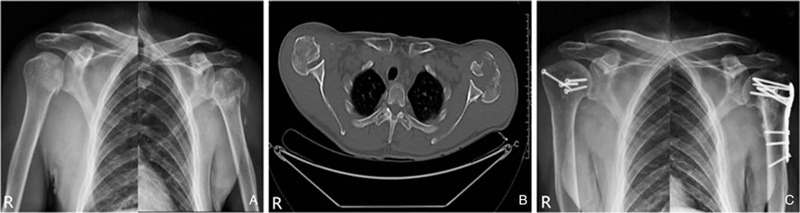
(a) Anteroposterior radiograph of the shoulders showing bilateral proximal humeral fractures; (b) axial CT of both sides confirming the dislocation and showing a posterior 4-part fracture-dislocation of the left shoulder and a posterior 2-part fracture-dislocation of the right shoulder; (c) postoperative X-ray showing good reduction of bilateral fractures and appropriate implants position.

A 55-year-old male was admitted to the ED for pain in upper limbs and shoulders after an electric shock. In the morning, while working in the vegetable shed, the patient accidentally contacted the high-voltage electricity with his right hand and was knocked down to the ground without loss of consciousness. Physical examination revealed that both upper limbs were in adduction and internal rotation position with marked movement limitations. Anteroposterior radiograph of the shoulders showed bilateral proximal humeral fractures (Fig. 2a). Neurovascular examination revealed injury to both axillary nerves. As there were multiple dermal burns in both upper limbs and the distal ends of the middle and little fingers were blackened, he was quickly transferred to burns department for debridement and amputation of fingers. As the patient was highly suspected of posterior fracture-dislocation of the shoulders, CT scan was performed the next day and the diagnosis was confirmed (Fig. 2b). There was a posterior 4-part fracture-dislocation of the left shoulder and a posterior 2-part fracture-dislocation of the right shoulder.^[7]^ According to the AO fracture classification, the left side was 11-C3.3 fracture and the right side was 11-C1.3 fracture. After comprehensive assessment, he was admitted to our orthopedics and traumatology for surgery. The ORIF with an angular stable plate (Philos, Synthes, Switzerland) was performed on the left side and ORIF with 4 screws was performed on the right side. Postoperative X-ray showed good reduction of bilateral fractures and appropriate implants position (Fig. 2c). He was discharged after 3 weeks of symptomatic supportive treatment. Both shoulders were fixed for 3 weeks postoperatively, followed by passive range of motion, active-assisted motion and progressively active exercises and rotator cuff strengthening exercises for the next 9 weeks. At 24 months follow-up, radiographs showed anatomically satisfactory bony union without avascular necrosis. The final Constant score^[14]^ was 69 on the left side and 71 on the right side. Both shoulders were painless and stable with acceptable range of motion and he was able to carry out daily activities.

## 3 Discussion

Bilateral posterior fracture-dislocation of the shoulder was first reported by Mynter in 1902,^[5]^ which is more common in middle-aged males.^[15]^ It is very rare and frequently misdiagnosed clinically.^[16]^ So far, research on this injury has been limited to case reports and small case series. It is a relatively difficult clinical challenge that requires systemic examination and evaluation and careful management planning.

### 3.1 Pathogenesis and cause

The pathogenesis of this posterior dislocation is considered to be the intense involuntary contraction of the muscles around the shoulder and the strength of internal rotators and adductors are greater than that of the external rotators and abductors, this unbalanced contraction creates a sustained tension that causes an excessive adduction and internal rotation, leading to a posterior dislocation.^[17]^

Shaw first clearly described the pathogenesis of this injury in 1971,^[11]^ who believed that in the setting of a seizure, the typical position of shoulder joint is flexion, adduction, and internal rotation. In this position, the muscles around the shoulder contract excessively and unbalancedly which forces the humeral head to be pulled out of the glenoid cavity and move posteriorly and superiorly.^[11,18]^ After the seizure, the humeral head is locked behind the glenoid. If epilepsy persists, continuing pressure against the glenoid rim can eventually lead to a complex proximal humeral fracture.^[3]^

The main causes of posterior shoulder dislocation described in the literature were summarized by Brackstone as “triple E syndrome,” which comprises epilepsy (or any convulsive seizure), electric shock (including electroconvulsive therapy), and extreme trauma (such as falls against outstretched arms and motor vehicle accidents).^[5]^ Almost 50% of bilateral posterior dislocations are caused by epilepsy, and this proportion rises to 90% if fracture occurs concomitantly, thus epilepsy is the most common cause.^[3]^ Other rare causes such as osteoporosis,^[19]^ stroke,^[20]^ hypoglycemic episodes,^[21]^ etc have also been reported. Pushpakumara J et al even reported that forceful suppression of the upper limbs in seizures may be the cause of the posterior fracture-dislocation of the shoulders for their patient.^[22]^ The 2 cases we report here are also very rare, one caused by a simple fall and the other by electrocution. Knowledge of the rare causes of this injury and a thorough clinical examination will help prevent missed diagnosis, whether from the perspective of orthopedics or neurologists, is essential for better planning of treatment.

### 3.2 Clinical manifestation

Posterior dislocation of the shoulders can be divided into 3 types: subacrominal type (most common), subglenoid type, and subspinous type, which is frequently accompanied by an impression defect of anteromedial articular surface of the humeral head caused by the impaction of the posterior glenoid rim into the head. As the pathogenesis is similar to the more common Hill-Sachs lesion in anterior dislocation, it is commonly called “reversed Hill-Sachs lesion” or “McLaughlin lesion.”^[23,24]^

The clinical presentations are severe pain and deformity around shoulder joints, often accompanied by anterior bruising of the upper arms. On physical examination, both arms are commonly locked in flexion, adduction, and internal rotation with severe limitation of external rotation even to neutral, which are characteristics of this entity. However, if associated fractures are found, this typical presentation may sometimes be masked, leading to missed diagnosis and dislocation of the shoulder, and many types of fractures, ranging from an impaction fracture of anteromedial humeral head to complex fractures in the proximal humerus. Two-part fractures of lesser tubercle, 2-part fractures of the anatomical neck, and complex 3- and 4-part fractures are the 3 most common types of fractures.^[15]^

If the patient has signs of locked internal rotation deformity, loss of normal contour of shoulders, increased prominence of the coracoid, palpable protuberance of humeral head at the back of the shoulder, obvious loss of external rotation, the possibility of posterior dislocation of shoulders should be highly suspected.^[25,26]^ At this time, detailed medical history inquiry and appropriate radiologic examination are essential for correct diagnosis.

### 3.3 Radiologic examination

Appropriate joint imaging is crucial for patients who suspected posterior dislocation of shoulder.^[27]^ The anteroposterior (AP) radiographs of the shoulders are easy to complete, but they may appear normal, thus contribute to a high misdiagnosis rate. The abnormal signs on AP view, such as light bulb sign, double shadow line sign, ice cream cone sign, rim sign, trough line sign, vacant glenoid sign, and so on, though have been described, are very rare.^[28,29]^

An axillary radiograph is essential for confirming the diagnosis. In a group of 40 patients, Hawkins et al^[12]^ found only a 50% diagnostic rate using AP and scapula lateral radiographs, and the rate increased to 100% after adding axillary radiograph. However, axillary views are often difficult to obtain in the acute situation due to pain and limited external rotation of the humerus. In such cases, modified axillary views (either a Velpeau or Wallace view) should be taken,^[26,30]^ and they are superior to axillary view because they can be taken even when the patient's arm is in the sling. CT is one of the most accurate imaging techniques for assessing bone injury. It provides more information about fractures than conventional X-rays. Therefore, if we consider surgical treatment, CT images of the shoulders are needed to help establish a precise treatment plan.^[31]^ CT can better visualize of the fractured region, evaluate the size of the defect of the articular surface of the humeral head, and its relation with the glenoid.^[32,33]^ Fracture fragments and their alignment as well as additional fracture lines can also be demonstrated.^[31]^

Ultrasound is a nonradioactive, inexpensive, and effective technique to identify posterior shoulder dislocation. As for infants and children, it may be better than conventional imaging. However, it cannot assess the associated bone damage.^[12]^

In a word, we should pay attention to appropriate radiographs, especially the axillary or the modified axillary views. CT image could provide an excellent visualization of fractured region, and is essential for planning surgery.^[34]^

### 3.4 Management

The posterior dislocation of the shoulder is very rare and there is a lack of consensus on current treatments.^[35]^ Several treatment options are available such as open or closed reduction, hemiarthroplasty, or total shoulder arthroplasty, etc. Much more attention should be paid to posterior dislocation of shoulder with fracture, while such patients are likely to require open reduction with fixation of the fracture, although closed reduction can be attempted. Thus it is suggested that orthopedic surgeon should be consulted as early as possible.^[18,36]^

The size of the defect in the humeral head and dislocation duration are the 2 critical points for treatment options. If the defect of humeral head articular surface is up to 25% and duration of dislocation is less than 3 weeks, closed reduction or open reduction can be attempted.^[37]^ Furthermore, if there is residual instability, it can be enhanced by subscapularis transfer.^[9,38]^ However, for chronic dislocations over 3 weeks, closed reduction is almost impossible and surgery is usually required,^[12,32,39]^ and if the humeral head is viable, open reduction and soft tissue release can be attempted. For a medium defect of 25% to 50% for the articular surface, the management are even more challenging,^[40]^ which may require reconstruction, McLaughin's or modified McLaughin's procedure, rotational osteotomy, etc, while for cooperative and especially muscular patients, closed management can also be attempted.^[41]^ For defects larger than 50%, surface replacement arthroplasty or hemiarthroplasty are the choice methods.^[42–44]^

Age is also an important factor for treatment decisions. Young patients with acute displaced fractures can try closed reduction, if unsuccessful, open reduction and internal fixation can be attempted. In elderly patients over 65 years old with 3 or 4 part fractures, because the high risk of humeral head avascular necrosis, the preferred treatment option is hemiarthroplasty.^[45]^ Total shoulder arthroplasty should be considered if head of humerus and glenoid cavity are damaged.^[46]^

Despite reports of the high incidence of avascular humeral head necrosis for the fracture types of our patients after ORIF,^[47,48]^ we still gave osteosynthesis a chance according to the relatively young age and good physical conditions of our 2 patients. Fortunately, neither of our patients has experienced necrosis so far and the good results justify our decision.

Therefore, the management should be individualized and multifactor should be considered, including the type of lesions, the size of the defect in the head of humerus, duration of dislocation, age, occupation, economic conditions, physical condition, and patient demands.^[4,5,37]^

## 4 Conclusion

Bilateral posterior fracture-dislocation of the shoulders rarely occurs and is often missed or delayed, leading to chronic pain, disability, and rising medical costs. Timely diagnosis and proper treatment are very important to restore shoulder function. One way to prevent missing diagnosis is to suspect cases with pain and limited external rotation, especially those with a history of seizures, electric shock, or severe trauma. Appropriate history inquiry and physical examination, as well as proper shoulder images such as axillary or modified axillary views and even CT scan of suspected cases, are the key to correct diagnosis. Treatment should be individualized and early consultation and evaluation by orthopedic surgeon is recommended.

## Acknowledgments

The authors are grateful to the patients and their families.

## Author contributions

**Conceptualization:** Bomin Wang, Shihong Xu.

**Formal analysis:** Junwei Wu, Fengrui Wang.

**Methodology:** Lianxin Li, Shun Lu, Honglei Jia.

**Supervision:** Lianxin Li, Fu Wang, Zhenhai Hao.

**Writing – original draft:** Junwei Wu.

**Writing – review & editing:** Fanxiao Liu, Yongliang Yang.
